# Implementation of a Package of Emergency Care Interventions and Clinical Outcomes

**DOI:** 10.1001/jamanetworkopen.2025.39471

**Published:** 2025-10-27

**Authors:** Corey B. Bills, Paul Wesseh, Catherine Cooper, Taylor W. Burkholder, Lane Epps, Michael Jaung, Emilie J. Calvello Hynes, Alex Mayah, John K. Shakpeh, Kayla Enriquez

**Affiliations:** 1 Department of Emergency Medicine, University of Colorado School of Medicine, Aurora; 2 Redemption Hospital, Ministry of Health, Monrovia, Liberia; 3 Ministry of Health, Monrovia, Liberia; 4 Department of Emergency Medicine, University of Southern California, Los Angeles; 5 Department of Emergency Medicine, University of California, San Francisco; 6 Department of Emergency Medicine, Baylor College of Medicine, Houston, Texas

## Abstract

**Importance:**

Investments in emergency care systems are vital to ensuring universal health coverage and improving health outcomes in low- and middle-income countries.

**Objective:**

To assess whether a package of emergency care interventions is associated with improved patient mortality and clinical care quality.

**Design, Setting, and Participants:**

This pre-post quality improvement study was conducted at a single urban referral hospital emergency unit (EU) in Monrovia, Liberia, to assess clinical and educational outcomes resulting from the implementation of a package of interventions from January 1, 2018, through June 30, 2019. Final analysis was performed in November 2023. Data from a random subset of adult patient encounters were collected retrospectively for the 12 months and compared with all adult patient presentations to the EU during the 6-month program implementation.

**Interventions:**

Triage, standardized documentations, and clinical teaching via a formal curriculum and bedside clinical mentorship.

**Main Outcomes and Measures:**

The primary outcome was all-cause mortality within 24 hours. Secondary outcomes included mortality at 48 hours, in-EU mortality, and EU quality process indicators. Multivariable logistic regression models were constructed to compare the association between program implementation and all-cause mortality.

**Results:**

A total of 344 preimplementation patients were compared with 1073 patients enrolled during the program with largely similar baseline characteristics between the 2 groups (mean [SD] age, 41.4 [16.4] vs 40.1 [17.3] years: 178 [51.7%] male and 164 [47.7%] female vs 601 [56.0%] male and 472 [44.0%] female; and 163 [47.3%] vs 510 [47.5%] near a hospital). All-cause mortality at 24 and 48 hours was significantly different between the preimplementation and implementation periods (27 [8.3%] vs 40 [3.9%], *P* < .001, and 34 [10.4%] vs 52 [5.0%], *P* < .001, respectively). In-EU mortality was significantly different between the 2 groups (13.5% [44 of 327] vs 7.1% [73 of 1031], *P* < .001). In multivariable regression, the adjusted odds of death at both 24 and 48 hours among patients in the intervention period was half that of the preintervention period.

**Conclusions and Relevance:**

This quality improvement study provides evidence that a set of interventions is associated with improved emergency care quality and reduced mortality. The high rates of EU-based mortality suggest the critical need to include EC in all facility-based quality improvement efforts.

## Key Points

**Question:**

Is a package of quality improvement interventions associated with improved clinical outcomes in a resource-limited setting in Liberia?

**Findings:**

This quality improvement study of 344 preimplementation patient encounters compared with 1073 enrolled during program implementation found that implementation of a package of low-cost interventions was associated with improvements in emergency care quality and significant reductions in early death.

**Meaning:**

These results suggest that prehospital and emergency unit mortality in this setting accounts for a substantial proportion of overall mortality in the health system, and low-cost interventions have the potential to lead to significant immediate and sustained improvements in both emergency unit quality and patient mortality.

## Teaser

This quality improvement study assesses whether the implementation of a package of emergency care interventions, as piloted at a single hospital in Liberia, was associated with improved clinical care quality.

## Introduction

Emergency conditions contribute to a substantial proportion of global deaths and disability, with a disproportionate burden occurring in low- and middle-income countries (LMICs).^1,2^ Estimates suggest that strengthening health systems to provide emergency care (EC) would address a large proportion of yearly deaths.^3,4^ Prior World Health Assembly resolutions advocated for strengthening and integration of EC in larger health systems to achieve universal health care.^5,6^ In tandem with policy directives, the World Health Organization (WHO) has developed several open-source tools to support improvements in EC quality globally.^7,8^

Previous studies on these tools have supported local acceptance and feasibility, improvements in participant knowledge-based test performance, and increased health care worker confidence.^9,10,11^ Detailed evidence of the value of a package of EC interventions in health facilities remains understudied, particularly data inclusive of clinical process measures and patient outcomes. The aim of this study was to assess whether the implementation of a package of EC interventions, as piloted at a single hospital in Liberia, was associated with improved clinical care quality.

## Methods

In this quality improvement study, we performed a pre-post quality improvement project to assess clinical outcomes associated with implementation of a package of facility-based EC interventions during a 6-month period from January 1, 2019, to June 30, 2019. The study was approved by the National Research Ethics Board of Liberia and reviewed and exempted by the institutional review board of the University of California, San Francisco. We adhered to Standards for Quality Improvement Reporting Excellence (SQUIRE) reporting guidelines during the study.^12^

### Setting

This study was conducted at Redemption Hospital, a public referral facility in Monrovia, Liberia. The adult emergency unit (EU) consists of approximately 10 beds with an associated 5-bed isolation unit.^13^ Redemption Hospital was strongly affected by Ebola, closed for a period and reopened after extensive decontamination, renovation, and development of infection, prevention, and control practices.^14^ Despite improvements, critical gaps in infrastructure, equipment, and human resources remained, leading to the design and implementation of this study.

### Study Design

We were guided in our work by several implementation frameworks.^15,16^ All EU frontline clinical practitioners (N = 56) were targeted and verbally consented for involvement. Consistent with most health practitioners in Liberia, none of the enrolled practitioners completed prior formal training in EC.

The package of EC interventions included several WHO tools: Interagency Triage Tool, designation of a bed for high-acuity patients, standardized clinical documentation forms, and a formal curriculum consisting of didactic lectures. The full package of interventions and associated sources is provided in eTable 1 in Supplement 1. We did not introduce decision rules beyond those described in the lecture material. Although space was reorganized to support triage and a dedicated resuscitation bed, no additional staffing, monitoring, or testing was made available to the EU during the study.

### Data Collection

We retrospectively sampled patient encounters for 12 months before program implementation from January 1, 2018, to December 31, 2018. A random number between 1 and 4 designated the first patient from the EU patient ledger followed by the enrollment of every fourth patient on the list thereafter. In the event a medical record was missing, the subsequent patient on the ledger was chosen until a medical record was obtained. Access to retrospective medical records and data extraction began on January 14, 2019.

Prospective medical record review began on January 1, 2019, and coincided with the end of the program on June 30, 2019. All patients 18 years or older presenting to and receiving care in the EU during the 6-month period were included in the study. Patients who left prior to being seen were excluded from the study. Paper medical records were collected, reviewed, and digitized with deidentified data extracted and stored in a secured, password-protected, online data storage platform via REDCap (Research Electronic Data Capture).^17^

### Outcomes 

Variables, including age, sex, village, chief concern (recategorized into larger descriptive fields),^18^ vital signs, patient history, and EU disposition, were collected and presented as number (percentage) or mean (SD). We compared pre-post process indicators of EU quality. Specific indicators, based on previous literature^19,20^ and with regard to local relevance, included timeliness to care (time: to EU after triage, from arrival to practitioner, from arrival to disposition, from arrival to antibiotics, and from arrival to intravenous fluids); recording of vital signs; intravenous fluids ordered in the setting of hypotension (defined as a systolic blood pressure <90 mm Hg) or elevated heart rate (>100 beats/min); tetanus vaccine ordered in traumatic injury; and antibiotics given to patients with presumptive infection as defined by positive Systemic Inflammatory Response Syndrome criteria.^21,22^

The primary outcome was all-cause mortality at 24 hours. Secondary outcomes of mortality included 48- and 72-hour mortality, location of death, and death by WHO-identified conditions sensitive to EC delivery: pneumonia, asthma, diabetic ketoacidosis, injury, pediatric diarrhea, and postpartum hemorrhage.^23^ Owing to diagnostic limitations and the lack of both pediatric and maternal patients seen in this setting, we included diarrhea, asthma, injury, lower respiratory tract infections, and blood glucose complications. Those dead before or on EU arrival were excluded from multivariable regression analyses.

### Statistical Analysis

Final analyses were performed in November 2023. A preimplementation sample size of 328 was calculated based on yearly EU census estimates, a presumed 7% absolute reduction of the primary outcome of all-cause 24-hour mortality assuming a 95% confidence level with a 5% margin of error. Pearson χ^2^ tests and Wilcoxon signed-rank sum tests were used to assess categorical and continuous variables, respectively. Univariable logistic regression was used to explore the association of individual variables with 24- and 48-hour mortality. We also tested for confounding variables through calculation of crude and variable-adjusted (Mantel-Haenszel) combined risk ratios. We constructed multiple multivariable logistic regression models for the outcome of mortality at 24 and 48 hours. Independent variables were chosen from known historical, demographic, and clinical risk factors for EU death in LMICs, known surrogates of critical illness, and from univariable analysis if *P* < .10. The number of variables used in final models was based on the number of absolute deaths at each time point. We reported unadjusted and adjusted odds ratios (ORs) and 95% CIs for independent variables to the outcome variables. The threshold for statistical significance was a 2-sided *P* < .05 in all analyses. Statistical analyses were performed using Stata SE, version 14.2 (StataCorp).

## Results

A total of 344 preimplementation patient encounters were compared with 1073 enrolled during program implementation (Table 1), with preimplementation patients similar to implementation patients in mean (SD) age (41.4 [16.4] vs 40.1 [17.3] years), sex (178 [51.7%] male and 164 [47.7%] female vs 601 [56.0%] male and 472 [44.0%] female), and proximity to the health facility (163 [47.3%] vs 510 [47.5%]). A broad number of EU quality indicators improved during implementation (Table 2). Recording of blood pressure (183 [56.0%] vs 756 [73.3%], *P* < .001) and oxygen saturation (0 vs 321 [31.1%]) both improved significantly. Documentation of temperature, heart rate, and respiratory rate remained unchanged, although practitioners acted more often on abnormal vital signs and therapeutics, such as intravenous fluids (14 [73.7%] vs 121 [91.0%], *P* = .04) when indicated by low blood pressure.

**Table 1.** Baseline Characteristics of Patients Before and During Implementation

Characteristic	No. (%) of patients				
	Before implementation (n = 344)	During implementation			
		Total (n = 1073)	Months 1-2 (n = 330)	Months 3-4 (n = 392)	Months 5-6 (n = 351)
Age, mean (SD), y	41.4 (16.4)	40.1 (17.3)	40.1 (16.7)	39.9 (17.6)	40.3 (17.6)
Sex					
Female	164 (47.7)	472 (44.0)	143 (43.3)	175 (44.6)	154 (43.9)
Male	178 (51.7)	601 (56.0)	187 (56.7)	217 (55.3)	197 (56.1)
Location, km					
<5	163 (47.4)	510 (47.5)	163 (49.4)	170 (43.4)	177 (50.4)
>5	152 (44.2)	462 (43.1)	151 (45.8)	162 (41.3)	149 (42.5)
Referred from other hospital					
No	NR	1037 (96.6)	329 (99.7)	383 (97.7)	329 (93.7)
Yes	NR	36 (3.4)	1 (0.3)	9 (2.3)	26 (7.4)
Day of week					
Weekday	268 (77.9)	778 (72.5)	229 (69.4)	300 (76.5)	249 (70.9)
Weekend	76 (22.1)	295 (27.5)	101 (30.6)	92 (23.5)	102 (29.1)
Time of day					
6:00 am to 1:59 pm	164 (47.7)	494 (46.0)	152 (46.1)	173 (44.1)	169 (48.1)
2:00 pm to 9:59 pm	106 (30.8)	326 (30.4)	98 (29.7)	108 (27.6)	120 (34.2)
10:00 pm to 5:59 am	50 (14.5)	171 (15.9)	48 (14.5)	77 (19.6)	46 (13.1)
Chief concern					
Abdominal pain	43 (12.5)	212 (19.8)	50 (15.2)	80 (20.4)	82 (23.4)
Change in mentation	26 (7.6)	157 (14.6)	37 (11.2)	59 (15.1)	61 (17.4)
Chest pain or palpitations	9 (2.6)	46 (4.3)	9 (2.7)	16 (4.1)	21 (6.0)
Diarrhea	9 (2.6)	67 (6.2)	19 (5.8)	25 (6.4)	23 (6.6)
Fever	12 (3.5)	103 (9.6)	30 (9.1)	36 (9.2)	37 (10.5)
Generalized weakness	45 (13.1)	225 (21.0)	50 (15.2)	98 (25.0)	77 (21.9)
Genitourinary concern	14 (4.1)	55 (5.1)	13 (3.9)	25 (6.4)	17 (4.8)
Injury and trauma	52 (15.1)	224 (20.9)	71 (21.5)	95 (24.2)	58 (16.5)
Nausea or vomiting	15 (4.4)	108 (10.1)	20 (6.1)	52 (13.3)	36 (10.3)
Shortness of breath or cough	25 (7.3)	90 (8.4)	21 (6.4)	41 (10.5)	28 (8.0)
Other or not recorded	94 (27.3)	210 (19.5)	81 (24.5)	63 (16.1)	66 (18.8)
Initial emergency disposition					
Admit^a^	32 (9.3)	196 (18.3)	88 (26.7)	72 (18.4)	36 (10.3)
Discharge	231 (67.2)	721 (67.2)	197 (59.7)	258 (65.8)	266 (75.8)
EU death or dead on EU arrival^b^	61 (17.7)	99 (9.2)	31 (9.4)	35 (8.9)	33 (9.4)
Left against medical advice	9 (2.6)	9 (0.8)	4 (1.2)	2 (0.5)	3 (0.9)
Transfer	11 (3.2)	48 (4.5)	10 (3.0)	25 (6.4)	13 (3.7)

Abbreviations: EU, emergency unit; NR, not reported.

^a^ Includes admitted patients who later died (6 in the before implementation group and 37 in the during implementation group).

^b^ Before disposition from the EU.

**Table 2.** Measures of Emergency Unit Quality Before and During Implementation

Characteristic	No. (%) of patients^a^					*P* value
	Before implementation (n = 327)	During implementation				
		Total (n = 1031)	Months 1-2 (n = 317)	Months 3-4 (n = 383)	Months 5-6 (n = 33)	
Vital signs recorded						
Blood pressure	183 (56.0)	756 (73.3)	207 (65.3)	217 (56.7)	205 (61.9)	<.001
Heart rate	177 (54.1)	537 (52.1)	182 (57.4)	182 (47.5)	173 (52.3)	.62
Oxygen saturation^b^	0	321 (31.1)	71 (22.4)	146 (38.1)	104 (31.4)	NA
Respiratory rate	159 (48.6)	463 (44.9)	169 (53.3)	157 (41.0)	137 (41.4)	.32
Temperature	298 (91.1)	865 (83.9)	259 (81.7)	309 (80.7)	297 (89.7)	.08
Temperature, heart rate, and respiratory rate	157 (48.0)	409 (39.7)	151 (47.6)	126 (32.9)	132 (39.9)	.01
Intravenous fluids given	135 (39.2)	544 (52.8)	134 (42.3)	220 (57.4)	190 (57.4)	<.001
If heart rate >100 beats/min^c^	47 (61.8)	115 (69.7)	30 (55.6)	45 (73.8)	40 (80.0)	.23
If SBP <90 mm Hg^d^	14 (73.7)	121 (91.0)	23 (82.1)	50 (92.6)	48 (92.3)	.04
Injury	57 (17.0)	224 (21.7)	71 (22.4)	95 (24.8)	58 (17.5)	.64
Tetanus given	17 (29.8)	152 (67.9)	50 (70.4)	52 (54.7)	32 (55.2)	<.001
Antibiotics given	228 (69.7)	712 (69.0)	189 (59.6)	246 (64.2)	174 (52.6)	.82
SIRS	89 (27.2)	111 (10.8)	31 (9.8)	41 (10.7)	39 (11.8)	<.001
Antibiotics if SIRS	68 (76.4)	82 (73.8)	21 (67.7)	26 (63.3)	20 (51.3)	.08

Abbreviations: NA, not applicable; SBP, systolic blood pressure; SIRS, systemic inflammatory response syndrome.

^a^ Excluding those who died before or on emergency unit arrival.

^b^ Fisher exact test.

^c^ Of those with documented heart rate (76 [42.9%] before implementation and 165 [30.7%] during implementation).

^d^ Of those with documented blood pressure (19 [10.4%] before implementation and 133 [17.6%] during implementation).

Improvements in measures of timeliness (including time to practitioner assessment, overall EU length of stay, and time to antibiotics and intravenous fluids) were largely immediate in the first 1 to 2 months and sustained through 6 months (Table 3). Although more patients were admitted to the hospital during program implementation (32 [9.3%] vs 196 [18.3%], *P* < .001), the length of hospital stay was significantly reduced (9.0 [11.1] vs 4.6 [5.4] days, *P* < .001).

**Table 3.** Comparison of EU Timeliness Before and During Implementation

Characteristic	Patients before implementation, mean (SD)^a^	Patients during implementation, mean (SD)^a^				*P* value
		Total	Months 1-2	Months 3-4	Months 5-6	
Time from triage^b^						
To EU, min	NR	12.8 (40.3)	13.6 (31.3)	17.8 (59.7)	7.8 (27.5)	NA
To practitioner, min	101.7 (202.2)	52.4 (75.5)	52.9 (74.9)	50.6 (64.9)	53.4 (84.8)	<.001
To antibiotics, min	343.5 (410.2)	204.2 (225.6)	213.4 (251.6)	188.6 (202.7)	218.4 (231.6)	<.001
To intravenous fluids, min	337.0 (431.5)	162.2 (218.2)	167.5 (242.4)	175.2 (228.8)	135.3 (162.5)	<.001
To disposition, h	103.0 (177.4)	39.6 (87.4)	45.6 (103.0)	44.1 (91.7)	25.7 (51.1)	<.001
Time to discharge, min	83.8 (167.7)	39.2 (83.7)	43.9 (95.0)	44.8 (91.9)	27.4 (55.3)	<.001
Time to admission, min	161.1 (229.7)	51.3 (112.8)	56.3 (135.3)	55.2 (105.1)	29.4 (41.5)	<.001
Total EU and hospital stay, d^c^	9.0 (11.1)	4.6 (5.4)	6.4 (6.2)	2.3 (3.0)	4.6 (5.4)	.005

Abbreviations: EU, emergency unit; NA, not applicable.

^a^ Excluding those who died before or on EU arrival.

^b^ Total sample sizes for the before implementation and during implementation groups were 194 and 342 for the time to practitioner, 98 and 177 for the time to antibiotics, 78 and 143 for the time to intravenous fluids, and 168 and 644 for the time to disposition, respectively.

^c^ Among patients admitted to the hospital from the EU.

The numbers (percentages) of those who died before or on EU arrival were similarly high (17 [25.8%] vs 42 [30.9%], *P* = .41). Most deaths occurred in the EU in both periods (44 of 66 [66.7%] and 73 of 136 [53.6%], respectively; *P* = .39), whereas the in-EU mortality rate (13.5% [44 of 327] vs 7.1% [73 of 1031], *P* < .001) was significantly lower after implementation (Table 4).

**Table 4.** Mortality Outcomes Before and During Implementation

Outcome	No. (%) of patients					*P* value
	Before implementation (n = 344)	During implementation				
		Total (n = 1073)	Months 1-2 (n = 330)	Months 3-4 (n = 392)	Months 5-6 (n = 351)	
Overall mortality	66 (19.2)	136 (12.7)	49 (14.8)	49 (12.5)	38 (10.8)	.003
Mortality proportional to time of death						
Before or on EU arrival	17 (25.8)	42 (30.9)	13 (26.5)	9 (18.4)	20 (52.6)	.39
<12 h	15 (22.7)	20 (14.7)	6 (12.2)	11 (22.4)	3 (7.9)	
12-<24 h	12 (18.2)	20 (14.7)	9 (18.4)	6 (12.2)	5 (13.2)	
24-<48 h	7 (10.6)	12 (8.8)	4 (8.2)	6 (12.2)	2 (5.3)	
48-72 h	4 (6.1)	5 (3.7)	2 (4.1)	3 (6.1)	0	
>72 h	11 (16.7)	37 (27.2)	15 (30.6)	14 (28.6)	8 (21.1)	
Cumulative mortality rate						
Before EU arrival	17 (4.9)	42 (3.9)	13 (3.9)	9 (2.3)	20 (5.7)	.41
<24 h	27 (8.3)	40 (3.9)	15 (4.7)	17 (4.4)	8 (2.4)	.001
<48 h	34 (10.4)	52 (5.0)	19 (6.0)	23 (6.0)	10 (3.0)	.001
<72 h	38 (11.6)	57 (5.5)	21 (6.6)	26 (6.8)	10 (3.0)	<.001
All	49 (15.0)	94 (9.1)	36 (11.4)	40 (10.4)	18 (5.4)	.003
Location of death						
Before or on EU arrival	17 (4.9)	42 (3.9)	13 (3.9)	9 (2.3)	20 (5.7)	.41
EU	44 (13.5)	73 (7.1)	23 (7.3)	34 (8.9)	16 (4.8)	<.001
Inpatient or transfer	5 (1.5)	21 (2.0)	13 (13.3)	6 (6.2)	2 (4.1)	.66
Death based on disposition						
EU-based deaths^a^	45 (15.3)	57 (6.8)	18 (7.9)	26 (8.4)	13 (4.4)	<.001
Ward admissions^b^	4 (12.5)	37 (18.9)	18 (20.5)	14 (19.4)	5 (13.9)	.38

Abbreviation: EU, emergency unit.

^a^ Among EU-based patients (not inclusive of those who died before or on EU presentation and not including those admitted; 295 before implementation and 835 during implementation).

^b^ Inclusive of admitted patients (regardless of location of death; 32 before implementation and 196 during implementation).

Preimplementation death rates were 2 times those during implementation at 24 hours (27 [8.3%] vs 40 [3.9%] for preimplementation vs implementation) and 48 hours (34 [10.4%] vs 52 [5.0%] for preimplementation vs implementation). Unadjusted all-cause mortality was significantly lower in the implementation vs preimplementation group, regardless of time (136 [12.7%] vs 66 [19.2%], *P* < .001).

Mortality rates were generally sustained throughout all subperiods of program implementation and across subcategories of time and place (eTable 2 in Supplement 1). Mortality at 24 hours was significantly reduced from the preimplementation to implementation periods among male patients (11.7% [20 of 171] vs 4.2% [24 of 574]), those who lived within 5 km of the hospital (9.5% [15 of 157] vs 3.9% [19 of 490]), and among those presenting during the weekdays (9.5% [24 of 254] vs 3.6% [27 of 755]; *P* < .001). In both the preimplementation and implementation periods, however, the number of patients diagnosed with malaria was similar (28 of 327 [8.6%] vs 91 of 1031 [8.8%]), as was the rate of death among patients with malaria (2 of 28 [7.1%] vs 7 of 91 [7.7%]).

Adjusting for age, sex, proximity to the health facility, and day of presentation did not suggest confounding (based on Mantel-Haenszel crude risk ratio analyses) to the overall risk of death in the implementation period. In univariable and multivariable logistic regression modeling, the odds of death in the implementation was halved, with less than 0.5 odds of death in the implementation period as compared with the preimplementation period at 24 hours (adjusted OR, 0.47; 95% CI, 0.26-0.83) and 48 hours (adjusted OR, 0.46; 95% CI, 0.28-0.76) (eTable 3 in Supplement 2).

## Discussion

Quality EC is a critical component of efforts to strengthen health systems globally. This study showed that a package of open access interventions can be implemented in a resource-limited setting and that such interventions are associated with significant improvements in clinical care. Specifically, implementation of a core set of EU-based interventions was associated with a 50% reduction in the odds of mortality at 24 and 48 hours—reductions in mortality found recently in preliminary studies^24,25,26^ of the WHO EC tools in Uganda and Ethiopia. In addition, reductions in EU-based mortality were significant regardless of the time of death, largely immediate on implementation, and sustained across the 6 months of the program. Lastly, when adjusted for multiple patient demographics, all-cause mortality in the implementation period remained lower.

This study also supports the feasibility of collecting data on a host of previously summarized EC quality indicators in LMICs.^18,27^ In this study, measures specific to timeliness, vital sign measurement, and increased intravenous fluid and antibiotic administration were able to be collected and improved during implementation. Additional studies on an agreed set of core EU quality indicators specific to LMICs, beyond mortality, would prove useful for comparison across different settings.

Although significant improvements in EU-based care were identified in this study, there remain several areas for ongoing improvement. First, most facility-based deaths continued to occur in the EU throughout the study period. Median EU mortality in several LMICs is many times higher than in high-income countries (1.8% vs 0.04%),^28,29,30,31,32,33^ with EU mortality at this study site even higher (4.9%). In addition to high EU mortality rates, more than 40 deaths (3.9%) occurred on or before EU arrival during the implementation period. Comparatively, the prevalence of patients dead on EU arrival among high-income countries is 0.15%.^34^ Additional studies on patient barriers to seek, reach, and receive EC in this context may prove helpful.^35^

### Limitations

This study has several important limitations. First, this was a single-site study adapted to the specific needs of the facility and undertaken just before the SARS-CoV-2 pandemic. Efforts to understand program sustainability and larger assessments of practitioner satisfaction with the intervention were not possible given many obstacles precipitated by COVID-19 and subsequent changes in local and national public health policies resulting in drastic changes in care-seeking behavior.

Second, similar to other work^36^ in LMICs, this study was limited to clinical paper medical records for data abstraction. Despite efforts to standardize sampling frames, inconsistent and missing records limited the quality of patient outcome data collected and may have introduced bias and the systematic inclusion of more severely ill patients in the preimplementation period. We have attempted to account for potential biases through presentation of baseline patient demographics (Table 1) and multivariable analyses aimed at controlling for patient-level variables. Among the variables included in the final regression model (eTable 3 in Supplement 1), weekend presentation was the only one with proportionally more patients in each group at baseline. In addition, ad hoc propensity scoring showed no difference between the proportionality of preconfounder and postconfounder variables used in the regression and the primary outcome of interest.

Third, seasonality (eFigure in Supplement 1) may also have affected death related to specific causes, such as malaria. In both the preimplementation and implementation periods, however, the number of patients diagnosed with malaria was similar (28 of 327 [8.6%] vs 91 of 1031 [8.8%]), as was the rate of death among patients with malaria (2 of 28 [7.1%] vs 7 of 91 [7.7%]). Relatedly, the study was not powered to detect differences among other high risk or core EU diagnoses, so it is not surprising that we did not detect significant improvements in these categories.

Fourth, this study may have a substantial risk of Hawthorne effect. Daily bedside clinical mentorship was intentional and served to encourage and model behavior change. Implementation of standardized clinical forms was also intentional and used as a method for data collection during implementation. Poor or no documentation in the preimplementation period among those with less severe concerns may also have led to an oversampling of critically ill patients during this period. It is unclear whether improvements in clinical quality in this context are owed to improvements in documentation, clinical care, or both. Future studies that directly observe practitioners may lead to better understanding of changes in care.

Fifth, we did not collect data specific to EU durable goods, supplies, and personnel; however, we were not made aware of significant changes during the study period. There was no known change in availability of resources, including no medication shortages or increased supply, and no additional durable goods were provided during the implementation period. Changes in clinical outcomes, such as the increase in measures of oxygen saturation, were not thought to have occurred as a result of improvements in access to medical supplies or increased clinical personnel. Despite these limitations, the general framework of this program is applicable to a wide variety of resource settings and is an important example of how basic tools can be incorporated into facility-based care with the intent to improve care.

## Conclusions

This quality improvement study provides evidence of the association of a set of interventions and improved EC quality and reduced mortality. The high rates of EU-based mortality highlight the critical need to include EC in all facility-based quality improvement efforts.

## Notes

Supplement 1. **eTable 1.** List of Tools Implemented During the Study Period and Source **eTable 2.** Overall Mortality, 24- and 48-Hour Mortality Stratified by Patient Characteristics and Diagnoses in the Pre and Implementation Period **eTable 3.** Odds of Death at 24 Hours, 48 Hours, and 30 Days **eFigure.** 24-Hour Mortality by Tertile

Supplement 2.
Data Sharing Statement
