# Supplementary material for: Implementation of a Package of Emergency Care Interventions and Clinical Outcomes
Source: JAMA Netw Open. 2025 Oct 27;8(10):e2539471. doi: 10.1001/jamanetworkopen.2025.39471 (PMC12559965; doi:10.1001/jamanetworkopen.2025.39471)
Supplement: Supplement 1. — eTable 1. List of Tools Implemented During the Study Period and Source eTable 2. Overall Mortality, 24- and 48-Hour Mortality Stratified by Patient Characteristics and Diagnoses in the Pre and Implementation Period eTable 3. Odds of Death at 24 Hours, 48 Hours, and 30 Days eFigure. 24-Hour Mortality by Tertile [file jamanetwopen-e2539471-s001.pdf]

Supplemental Online Content

Bills CB, Wesseh P, Cooper C, et al. Implementation of a package of emergency care interventions and clinical outcomes. *JAMA Netw Open*. 2025;8(4):e2539471. doi:10.1001/jamanetworkopen.2025.39471

eTable 1. List of Tools Implemented During the Study Period and Source
eTable 2. Overall Mortality, 24- and 48-Hour Mortality Stratified by Patient Characteristics and Diagnoses in the Pre and Implementation Period
eTable 3. Odds of Death at 24 Hours, 48 Hours, and 30 Days
eFigure. 24-Hour Mortality by Tertile

This supplemental material has been provided by the authors to give readers additional information about their work.

© 2025 Bills CB et al. *JAMA Network Open.*

eTable 1. List of Tools Implemented During the Study Period and Source

Title	Type	Month implemented	Source	Web link
WHO Basic Emergency Care Course	Didactic Teaching*	1-2	WHO**	https://www.who.int/publications/i/item/9789241513081
African Federation for Emergency Medicine Emergency Care Curriculum	Didactic Teaching*	2-6	AFEM	https://afem.africa/resources/
WHO Standardized Clinical Forms for Medical Patients	Standardized Clinical Form	1	WHO**	https://www.who.int/tools/clinical-checklists
WHO Standardized Clinical Forms for Trauma Patients	Standardized Clinical Form	1	WHO**	https://www.who.int/tools/clinical-checklists
Interagency Integrated Triage Tool	Triage	1	WHO**	https://www.who.int/tools/triage
Designating a Resuscitation Area in the emergency unit	Resuscitation Area	1	WHO**	https://www.who.int/publications/m/item/resuscitation-area-designation

*Didactic teaching included the WHO Basic Emergency Care course content and lectures from the African Federation of Emergency Medicine (AFEM) open-source lecture bank delivered via short, on shift clinical sessions held two to three times a day to minimize disruptions to patient care. Lectures and associated bedside teaching was facilitated by 9 visiting faculty over the 6-month time implementation period. Throughout the implementation period the ratio of clinical mentorship to frontline provider staffing was consistent, with a roughly 1:10 mentor-to provider ratio.

**Though not available at the time of this intervention, a full approach to Quality Improvement using the aforementioned WHO tools can be found at https://www.who.int/teams/integrated-health-services/clinical-services-and-systems/clinical-quality-improvement.

© 2025 Bills CB et al. *JAMA Network Open.*

eTable 2. Overall Mortality, 24- and 48-Hour Mortality Stratified by Patient Characteristics and Diagnoses in the Pre and Implementation Period

	All					24-hour					48-hour				
	Pre-implementation (N=327)		Implementation (N=1031)			Pre-implementation (N=327)		Implementation (N=1031)			Pre-implementation (N=327)		Implementation (N=1031)		
	Survived	Death	Survived	Death	P-value	Survived	Death	Survived	Death	P-value	Survived	Death	Survived	Death	P-value
Characteristic	N (%)	N (%)	N (%)	N (%)		N (%)	N (%)	N (%)	N (%)		N (%)	N (%)	N (%)	N (%)	
Total*	278 (85.0)	49 (15.0)	937 (90.9)	94 (9.1)	0.003	300 (91.7)	27 (8.3)	991 (96.1)	40 (3.9)	0.001	293 (89.6)	34 (10.4)	979 (95.0)	52 (5.0)	0.001
Sex															
Female	135 (87.7)	19 (12.3)	415 (90.8)	42 (9.2)	0.26	147 (95.5)	7 (4.5)	441 (96.5)	16 (3.5)	0.56	142 (92.2)	12 (7.8)	436 (95.4)	21 (4.6)	0.13
Male	141 (82.5)	30 (17.5)	522 (90.9)	52 (9.1)	0.002	151 (88.3)	20 (11.7)	550 (95.8)	24 (4.2)	<0.001	149 (87.1)	22 (12.9)	543 (94.6)	31 (5.4)	0.001
Proximity to health facility															
<5 km	131 (83.4)	26 (16.6)	443 (90.4)	47 (9.6)	0.02	142 (90.5)	15 (9.5)	471 ((95.1)	19 (3.9)	0.006	137 (87.3)	20 (12.7)	463 (94.5)	27 (5.5)	0.002
>5km	127 (87.6)	18 (12.4)	415 (92.8)	32 (7.2)	0.05	137 (94.5)	8 (5.5)	434 (97.1)	13 (2.9)	0.14	135 (93.1)	10 (6.9)	431 (96.4)	16 (3.6)	0.09
Time of Day															
6:00-13:59	138 (86.8)	21 (13.2)	434 (90.4)	46 (9.6)	0.20	149 (93.7)	10 (6.3)	460 (95.8)	20 (4.2)	0.27	146 (91.8)	13 (8.2)	455 (94.8)	25 (5.2)	0.17
14:00-21:59	87 (84.5)	16 (15.5)	289 (92.3)	24 (7.7)	0.02	95 (92.2)	8 (7.8)	304 (97.1)	9 (2.9)	0.03	92 (89.3)	11 (10.7)	301 (96.2)	12 (3.8)	0.008
22:00-5:59	39 (84.8)	7 (15.2)	155 (93.9)	10 (6.1)	0.04	41 (89.1)	5 (10.9)	160 (97.0)	5 (3.0)	0.04	41 (89.1)	5 (10.9)	158 (95.8)	7 (4.2)	0.14
Weekend															
Yes	65 (89.0)	8 (11.0)	254 (92.0)	22 (8.0)	0.42	70 (95.9)	3 (4.1)	263 (95.3)	13 (4.7)	1.00	68 (93.2)	5 (6.8)	262 (94.9)	14 (5.1)	0.56
No	213 (83.9)	41 (16.1)	683 (90.5)	72 (9.5)	0.004	230 (90.5)	24 (9.5)	728 (96.4)	27 (3.6)	<0.001	225 (88.6)	29 (11.4)	717 (95.0)	38 (5.0)	<0.001
High risk diagnoses**															
CVA	6 (60.0)	4 (40.0)	20 (60.6)	13 (39.4)	1.000	8 (80.0)	2 (20.0)	30 (90.9)	3 (9.1)	0.58	8 (80.0)	2 (20.0)	30 (90.9)	3 (9.1)	0.58
HIV/immunosuppression	3 (60.0)	2 (40.0)	13 (65.0)	7 (35.0)	1.000	3 (60.0)	2 (40.0)	19 (95.0)	1 (5.0)	0.09	3 (60.0)	2 (33.3)	18 (90.0)	2 (10.0)	0.17
Malaria	26 (92.9)	2 (7.1)	84 (92.3)	7 (7.7)	1.000	27 (96.4)	1 (3.6)	88 (96.7)	3 (3.3)	1.00	26 (92.9)	2 (7.1)	87 (95.6)	4 (4.4)	0.63
Sepsis	7 (43.75)	9 (56.25)	11 (44.0)	14 (56.0)	0.99	10 (62.5)	6 (37.5)	18 (72.0)	7 (28.0)	0.52	7 (43.8)	9 (56.2)	16 (64.0)	9 (36.0)	0.20
Core EU Diagnosis combined	103 (91.2)	10 (8.85)	364 (94.1)	23 (5.9)	0.27	108 (95.6)	5 (4.4)	377 (97.4)	10 (2.6)	0.346	107 (94.7)	6 (5.3)	372 (96.4)	14 (3.6)	0.42
Asthma	7 (100)	0 (0.00)	23 (100.0)	0 (0)	--	7 (100)	0 (0)	23 (100)	0 (0)	--	7 (100)	0.00	23 (100)	0.00	--
Diabetes complications	6 (66.7)	3 (33.3)	11 (61.1)	7 (38.9)	1.00	8 (88.9)	1 (11.1)	15 (83.3)	3 (16.7)	1.00	7 (77.8)	2 (22.2)	14 (77.8)	4 (22.2)	1.00
Diarrhea	25 (100)	0 (0.00)	59 (89.4)	7 (10.6)	0.18	25 (100)	0 (0)	63 (95.5)	3 (4.5)	0.56	25 (100)	0 (0.0)	61 (92.4)	5 (7.6)	0.33
Injury	55 (98.2)	1 (1.8)	248 (99.2)	2 (0.8)	0.46	55 (98.2)	1 (1.8)	248 (99.2)	2 (0.8)	0.46	55 (98.2)	1 (1.8)	248 (99.2)	2 (0.8)	0.46
Pneumonia	10 (62.5)	6 (37.5)	23 (76.7)	7 (23.3)	0.31	13 (81.3)	3 (18.7)	28 (93.3)	2 (6.7)	0.33	13 (81.3)	3 (18.7)	27 (90.0)	3 (10.0)	0.41

*Excluding OHCA: out of hospital cardiac arrest, including the majority of dead before arrival

**Fishers exact

***Post-partum hemorrhage and pediatric deaths were not included as there were no deaths attributable

Abbreviations: EU, Emergency Unite; CVA, Cerebral Vascular Accident; HIV, Human Immunodeficiency Virus

© 2025 Bills CB et al. *JAMA Network Open.*

eTable 3. Odds of Death at 24 Hours, 48 Hours, and 30 Days

	24-hour mortality		48-hour mortality		30 days	
Variable	Unadjusted OR (95% CI)	Adjusted OR (95% CI)	Unadjusted OR (95% CI)	Adjusted OR (95% CI)	Unadjusted OR (95% CI)	Adjusted OR (95% CI)
Implementation	0.45 (0.27-0.74)	0.47 (0.26-0.83)	0.46 (0.29-0.72)	0.46 (0.28-0.76)	0.57 (0.39-0.82)	0.58 (0.39-0.88)
Older age	1.50 (0.80-2.81)	1.44 (0.72-2.87)	1.54 (0.88-2.69)	1.50 (0.82-2.74)	1.78 (1.16-2.75)	1.70 (1.06-2.71)
Female Sex	1.60 (0.96-2.69)	1.61 (0.90-2.89)	1.34 (0.86-2.10)	1.31 (0.79-2.17)	1.12 (0.79-1.58)	1.08 (0.74-1.60)
Home within 5 km	0.66 (0.38-1.16)	0.62 (0.35-1.10)	0.59 (0.36-0.96)	0.56 (0.34-0.94)	0.73 (0.50-1.06)	0.73 (0.49-1.08)
Weekend	0.89 (0.50-1.57)	0.66 (0.31-1.37)	0.81 (0.48-1.37)	0.59 (0.30-1.14)	0.75 (0.49-1.14)	0.62 (0.37-1.02)

© 2025 Bills CB et al. *JAMA Network Open.*

eFigure. 24-Hour Mortality by Tertile

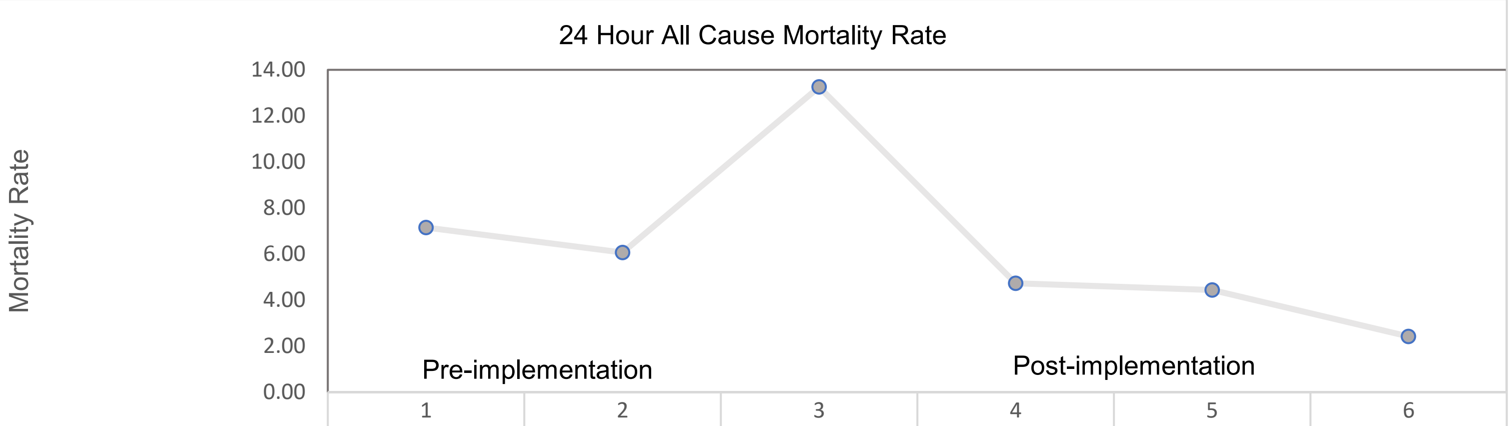

	1	2	3	4	5	6
24 Hour Mortality Rate	7.14	6.06	13.25	4.73	4.44	2.42

© 2025 Bills CB et al. *JAMA Network Open.*
