# Supplementary material for: Implementation of a Package of Emergency Care Interventions and Clinical Outcomes
Source: JAMA Netw Open. 2025 Oct 27;8(10):e2539471. doi: 10.1001/jamanetworkopen.2025.39471 (PMC12559965; doi:10.1001/jamanetworkopen.2025.39471)
Supplement: Supplement 2. — Data Sharing Statement [file jamanetwopen-e2539471-s002.pdf]

# Data Sharing Statement

Bills. Implementation of a Package of Emergency Care Interventions and Clinical Outcomes. *JAMA Netw Open*. Published October 27, 2025. doi:10.1001/jamanetworkopen.2025.39471

**Data**
**Data available:** Yes
**Data types:** Deidentified participant data
**How to access data:** Data will be made available via the following email address: corey.bills@cuanschutz.edu
**When available:** With publication

**Supporting Documents**
**Document types:** None

**Additional Information**
**Who can access the data:** Anyone requesting data
**Types of analyses:** For any purpose related to research or public health reasons
**Mechanisms of data availability:** With investigator support
